# Supplementary material for: NB-LRRs Not Responding Consecutively to Fusarium oxysporum Proliferation Caused Replant Disease Formation of Rehmannia glutinosa
Source: Int J Mol Sci. 2019 Jun 29;20(13):3203. doi: 10.3390/ijms20133203 (PMC6651281; doi:10.3390/ijms20133203)
Supplement: Supplementary file 1 [file ijms-20-03203-s001.pdf]

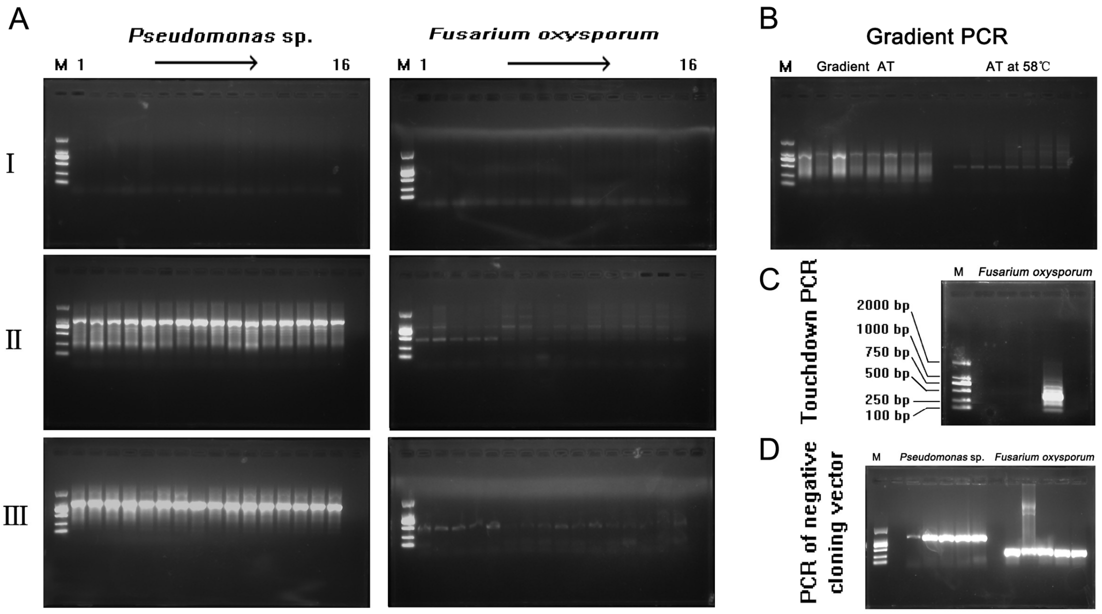

Figure S1. Electrophoretic strips of three DNA extraction methods for *Pseudomonas* spp. and *Fusarium oxysporum*, and optimizing annealing temperature using different PCR. A. The effect of three DNA extraction methods for *Pseudomonas* spp. and *Fusarium oxysporum*, the same row of which represents the same extraction method, and the same column represents the same microorganism. Method I referenced to Zhou et al. (1996). Methods II and III optimized humus removal. Annealing temperature was at 58 °C. B. Two PCR effects of 8 random soil DNA samples extracted by method Ⅲ (Left: gradient PCR with annealing temperature from 50 °C to 60 °C, eight gradients; Right: conventional PCR, 58 °C for annealing). C. Touchdown PCR effects of 1 random soil DNA samples extracted by method Ⅲ for FO. D. The electrophoretic strips of negative cloning vector PCR. Conventional PCR by ThermoFisher Scientific A24812, USA, gradient PCR by BIO-RAD T100TM, USA and touchdown PCR by BIO-RAD T100TM, USA. *AT* annealing temperature. *M* DL2000 Plus DNA Marker.

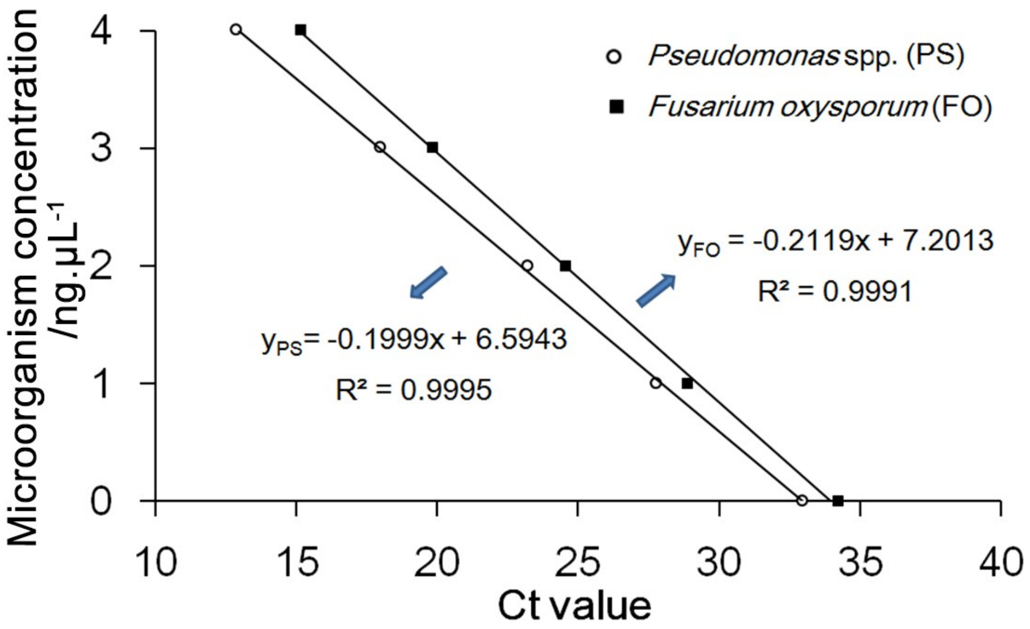

Figure S2. Standard curve of concentration and Ct value for *Pseudomonas* spp. and *Fusarium oxysporum*.

Table S1. Sequence of DNA fragments inserted into pMD19-T vector.

**microorganism name**	**Size of DNA fragments**	**Sequence (5′- 3′)**
Pseudomonas spp.	987 bp	gtctgagaggatgatcagtcacactggaactgagacacggtccagactctacgggaggcagcagtgg<br>gga<br>atattggacaatgggggcaaccctgatccagccatgccgcgtgtgtgaagaaggccttcgggttgtaa<br>ag<br>cactttaagttgggaggaaggctttaaggttaatatccttgaagattgacgttaccaacagaataagcac<br>cggctaactccgtgccagcagccgcggtaatacggagggtgcaagcgttaatcggaattactgggcgt<br>aa<br>agcgcgcgtaggcggccatttaagctagatgtgaaagcccagggcttaaccttggaactgcatttagaa<br>c<br>tggatggctagagtatggaagaggagtgtggaatttcaggtgtagcggtgaaatgcgtagagatctg<br>aag<br>gaacatcagtggcgaaggcgacactctggtccaatactgacgctgaggtgcgaaagcgtggggatca<br>aac<br>aggattagataccctggtagtccacgccgtaaacgatgtcgactagccgttggcctccttgaggggtta<br>g<br>tggcgcagctaacgcgataagtcgaccgcctggggagtacggccgcaaggttaaaactcaaatgaatt<br>ga<br>cgggggcccgcacaagcggtggagcatgtggtttaattcgacgcaacgcgaagaaccttacctactctt<br>g<br>acatccagataaccctgcagagatgtgggggtgccttcgggaaatctgagacaggtgctgcatggctg<br>tc<br>gtcagctcgtgtcgtgagatgttgggttaagtcccgtaacgagcgcaacccttgtccttagttgccagca<br>cgtaatggtgggaactctaaggagactgccggtgacaaaccggaggaaggtggggacgacgtcaag<br>tcat<br>catggcccttacgagtagggctacacacgtgctacaatggggagtacagagggaagcgaagccgcg<br>agtg<br>cagctaa
Fusarium oxysporum f.sp. *R. glutinosa*	433 bp	gcagctggcacgacaggtttcccgactggaaagcgggcagtgagcgcaacgcaattaatgtgagtta<br>gct<br>cactcattaggcaccccaggctttacactttatgcttccggctcgtatgttgtgtggaattgtgagcgga<br>taacaatttcacacaggaaacagctatgaccatgattacgccaagcttgcatgcctgcaggtcgacgatt<br>cttggtcatttagaggaagtaaaagtcgtaacaaggtctccgttggtgaaccagcggagggatcattac<br>c<br>gagtttacaactcccaaacccctgtgaacatacctatacgttgcctcggcggatcagcccgcgccctgta<br>aaaagggacggcccgcccgaggaccctaaactctgtttttagtggaacttctgagtaaaacaaacaaat<br>a<br>aatcaaaactttc

Table S2. Specific primer sequences of 35 *NB-LRRs* for qRT-PCR.

Gene name	Primer	Sequence (5′- 3′)
RgNB1	*RgNB1*-for	GAATCCTTCGGCTCAGAAACT
	RgNB1-rev	GCTTGGTGTGCTCATCTTC
RgNB2	*RgNB2*-for	AGGCACACCACCACACTTCTTC
	RgNB2-rev	CGTGCGTTTGGACAGGAGGAAA
RgNB3	*RgNB3*-for	TGATTGACCTTGGACAGAACTC
	RgNB3-rev	TGCCCTCGTGAACTGGAG
RgNB4	*RgNB4*-for	GCTACATCTCCTTCGACATACTCC
	RgNB4-rev	GCCCTTGCCGTGATTGGT
RgNB5	*RgNB5*-for	CTGAACTTCCGTCTTCGTTACTG
	RgNB5-rev	TCCAGATTGCTCCAACTCTCA
RgNB6	*RgNB6*-for	TTGCGTGAGATGCCTATT
	RgNB6-rev	TTCCCTGCCATAAACAAGA
RgNB7	*RgNB7*-for	GGTTGGATAAGTCGCTGTGTT
	RgNB7-rev	CAATGCTAGGTCTTGCCATAGG
RgNB8	*RgNB8*-for	TTCGCCGTCTTGAAGTGTT
	RgNB8-rev	CCGTCTAAGTCTAGGAAAGTGATG
RgNB9	*RgNB9*-for	ACCGTCGCCTTAGCATTCATT
	RgNB9-rev	TTCGGCAGAGAAGCACAAGA
RgNB10	*RgNB10*-for	TCGTTCCACCATTGCTCAA
	RgNB10-rev	ATCCTGCTGCTTCCTTCAC
RgNB11	*RgNB11*-for	GGCAGATGGTACATGAGAACA
	RgNB11-rev	GTCGTGGTAACTGAAGGCTAA
RgNB12	*RgNB12*-for	AATATGGATCTGGTGTCGTT
	RgNB12-rev	GCAATACCTCGGCATCTC
RgNB13	*RgNB13*-for	ATTCAACAACAGGTATCTCATT
	RgNB13-rev	CCAACTCTCATCTTCATTCAA
RgNB14	*RgNB14*-for	GCTCTGTGGAGAGTCATCTG
	RgNB14-rev	ATCATAAGCATACCTGGCAAGA
RgNB15	*RgNB15*-for	GATTTGCTTCGCCACGCTACG
	RgNB15-rev	GCAGAAGAGGAACACCATCACCAA
RgNB16	*RgNB16*-for	GATTATGCCTCGTCTCAG
	RgNB16-rev	TCCAATCCTCTCCATTCT
RgNB17	*RgNB17*-for	GGATCTTGCTGTTCTTGACCTCTC
	RgNB17-rev	CCTCCGTGAATATCCCACCATCT
RgNB18	*RgNB18*-for	GCATTCCAGTCCTCCACAC
	RgNB18-rev	ACGACTTAGCCGAGATGGT
RgNB19	*RgNB19*-for	AAGGCGTCAAGAATTGGACTGAT
	RgNB19-rev	GCGAAGGGCAAACGACTCA
RgNB20	*RgNB20*-for	CGAATGTAAGAAGAAGCAACC
	RgNB20-rev	TCAGCCAGAAGCAAACTT
RgNB21	*RgNB21*-for	CGTGAATTGGAAGGAGTTGGAAT
	RgNB21-rev	CGTTCGACTTTGGCTAGGATTC
RgNB22	*RgNB22*-for	AGTAATCATCCGCAACATCTTCCA
	RgNB22-rev	CGAGGCTGATATGGCTGTGG
RgNB23	*RgNB23*-for	CACTTGACACTATCGGATGG
	RgNB23-rev	TTGAGACGGATGGAGATTG
RgNB24	*RgNB24*-for	GCCAACGCCTACTATCACTT
	RgNB24-rev	TCATGCTGCCAACATAGAACTG
RgNB25	*RgNB25*-for	CTTCGCCTCCTCACTCAG
	RgNB25-rev	GCAAGCCAGATAGGGTCAG
RgNB26	*RgNB26*-for	TGCGGATTTGAGGGTCAGATTTCT
	RgNB26-rev	CGGACAACTCCCACCTTCCATATC
RgNB27	*RgNB27*-for	TCCTGCTCGTGTTAGATGACTACT
	RgNB27-rev	GCTTCCTCTTGACCCGACTTT
RgNB28	*RgNB28*-for	GCACTCACCACAGACGAATC
	RgNB28-rev	TGTAGCGAGAGTAGTCTTACCAAT

RgNB29	*RgNB29*-for	TCTCTTCCATACACATCAGACC
	RgNB29-rev	GACTCAACTTGCCGTTATCAG
RgNB30	*RgNB30*-for	CGGCGAATAAGTCTGTTACC
	RgNB30-rev	GTACCTTGGTGGATTAGTAAGC
RgNB31	*RgNB31*-for	GCTCAGCATCAAAGTAAGGAACA
	RgNB31-rev	CGTAAGTGTACTCAAGAGGAATGG
RgNB32	*RgNB32*-for	AGGCTGGTGAAGAGGTACTGTCA
	RgNB32-rev	TTGTGATGAGTGCCAGAGGTAGAC
RgNB33	*RgNB33*-for	TTGTTGAGTTGGTAGAGGTC
	RgNB33-rev	GTGGAGTTCTTGCTGGAG
RgNB34	*RgNB34*-for	GTGACTTACCATCTCCTTGT
	RgNB34-rev	AGCCCTTTAAGAACACCTTC
RgNB35	*RgNB35*-for	GTCCTTCTGCTACGGCTTCA
	RgNB35-rev	GGACATTGCCAGTGCCATCT
18S RNA	*18S RNA*-for	GAGCTAATACGTGCAACAAACC
	18S RNA-rev	CGAAAGTTGATAGGGCAGAAAT
